# Evaluating Normalization Methods for Robust Spectral Performance Assessments of Hyperspectral Imaging Cameras

**DOI:** 10.3390/bios15010020

**Published:** 2025-01-04

**Authors:** Siavash Mazdeyasna, Mohammed Shahriar Arefin, Andrew Fales, Silas J. Leavesley, T. Joshua Pfefer, Quanzeng Wang

**Affiliations:** 1 Center for Devices and Radiological Health, U.S. Food and Drug Administration, Silver Spring, MD 20993, USA; siavash.mazdeyasna@fda.hhs.gov (S.M.); mohammedshahriar.arefin@fda.hhs.gov (M.S.A.); andrew.fales@fda.hhs.gov (A.F.); joshua.pfefer@fda.hhs.gov (T.J.P.); 2 Chemical and Biomolecular Engineering, University of South Alabama, Mobile, AL 36688, USA; leavesley@southalabama.edu

**Keywords:** medical hyperspectral imaging, hyperspectral endoscopy, reflectance spectrum, normalization, centering, scaling, linear transformation, nonlinear transformation, bias, root mean square error, correlation coefficient

## Abstract

Hyperspectral imaging (HSI) technology, which offers both spatial and spectral information, holds significant potential for enhancing diagnostic performance during endoscopy and other medical procedures. However, quantitative evaluation of HSI cameras is challenging due to various influencing factors (e.g., light sources, working distance, and illumination angle) that can alter the reflectance spectra of the same target as these factors vary. Towards robust, universal test methods, we evaluated several data normalization methods aimed at minimizing the impact of these factors. Using a high-resolution HSI camera, we measured the reflectance spectra of diffuse reflectance targets illuminated by two different light sources. These spectra, along with the reference spectra from the target manufacturer, were normalized with nine different methods (e.g., area under the curve, standard normal variate, and centering power methods), followed by a uniform scaling step. We then compared the measured spectra to the reference to evaluate the capability of each normalization method in ensuring a consistent, standardized performance evaluation. Our results demonstrate that normalization can mitigate the impact of some factors during HSI camera evaluation, with performance varying across methods. Generally, noisy spectra pose challenges for normalization methods that rely on limited reflectance values, while methods based on reflectance values across the entire spectrum (such as standard normal variate) perform better. The findings also suggest that absolute reflectance spectral measurements may be less effective for clinical diagnostics, whereas normalized spectral measurements are likely more appropriate. These findings provide a foundation for standardized performance testing of HSI-based medical devices, promoting the adoption of high-quality HSI technology for critical applications such as early cancer detection.

## 1. Introduction

Medical hyperspectral imaging (HSI) has gained popularity because it provides both spatial information and a wide range of spectral data. This enables the characterization and quantification of structural and functional information of biological tissue [1,2,3,4], unlike traditional imaging cameras that offer only spatial information. In biological tissues, heterogeneity and disease can alter tissue properties, leading to changes in spectral information [2,5,6]. HSI cameras can capture these spectral variations across a wide wavelength range, aiding in surveillance for abnormal tissue. Therefore, HSI is expanding into different clinical applications, including cancer detection [2], surgical guidance [7], wound assessment [8], and endoscopy [9].

HSI data (i.e., a hypercube that includes both spatial and spectral information) can be collected using various methods. Whiskbroom (point-scanning) systems acquire spectral data while scanning across two spatial dimensions (x-y). Pushbroom (line-scanning) systems capture spatial information along one spatial direction and spectral information simultaneously, requiring scanning in the other spatial direction to form the hypercube. Spectral scanning systems acquire spatial data in each image and scan across a defined wavelength range using methods such as tunable filters with narrow bandwidths. Snapshot systems, by contrast, capture the entire hypercube in a single acquisition without the need for scanning. For an overview of different HSI technologies, please see [10].

Quantitative evaluation of HSI cameras is essential for ensuring accuracy and reliability in clinical procedures. It facilitates the comparison of devices and therefore is a key topic of industry standards. Establishing reliable evaluation methods will facilitate the refinement and advancement of HSI technologies, leading to improved effectiveness in critical health care roles. However, quantitative evaluation of HSI cameras is challenging due to the influence of device and measurement variables, which can cause the reflectance spectra of the same target or tissue to vary under otherwise similar conditions. These variables include measurement distance, light source spectrum, illumination intensity and uniformity [4], and ambient light fluctuations [11]. Enhancing robustness to these factors will improve a test method’s ability to assess an HSI camera’s capability in detecting true variations in the target or tissue. To reduce the impact of these factors, it is common to implement data normalization methods [12,13].

Previous studies have shown that normalizing spectral data enhances the performance of machine/deep learning techniques for analyzing HSI data [14]. Researchers have proposed various normalization methods that are widely used in data analysis and spectral image processing [12,13,14,15,16,17,18]. Cao et al. and Zhang et al. demonstrated the application of multiple normalization methods and highlighted their impacts on classification accuracy [14,16]. Singh et al. explored how classification accuracy and feature selection could be influenced by five different categories of normalization methods [17]. Witteveen et al. utilized eight commonly used preprocessing methods to reduce reflection artifacts (i.e., glare) from the collected and simulated tissue spectra [18]. Collectively, these studies suggest that normalization can significantly improve classification accuracy by enhancing data quality. However, there is still a lack of systematic studies that compare and quantify the performance of different normalization methods. Since the y-axis scale of normalized data (i.e., the “units” of the normalized reflectance values) can vary across methods, directly comparing results is challenging. While relative error measurements may be useful, some normalization methods include zero or near-zero reference values and thus can inflate relative errors. This highlights the need for a deeper understanding of how different normalization methods function when applied to HSI.

The purpose of the paper is to advance standardization of the performance evaluation of HSI systems. Specifically, we systematically evaluated and compared popular normalization methods by analyzing reflectance spectra collected from diffuse reflectance targets using a high-resolution HSI camera under two different light sources. Moreover, we analyzed the mathematical framework of these methods, identifying both their similarities and differences, and implemented a uniform scaling approach to compare their performance. The goal was to identify a robust normalization method that can mitigate the impact of external factors, thus enabling reliable evaluation of HSI systems.

## 2. Methodology

### 2.1. Capture of Reflectance Spectra with an HSI Camera

To evaluate spectral normalization methods, we measured HSI image data (hypercubes) from a Spectralon^®^ wavelength calibration target (WCS-EO-010. Labsphere, Inc., North Sutton, NH, USA) using an HSI camera (4250 VNIR, Hinalea Imaging Corp., Emeryville, CA, USA). This camera employs a Fabry–Perot Interferometer (FPI) to selectively transmit specific wavelengths of light. The FPI consists of two parallel, highly reflective mirrors that create multiple reflections, leading to constructive or destructive interference depending on the wavelengths. Only wavelengths satisfying the resonance condition pass through, enabling precise spectral filtering. By tuning the mirror spacing or refractive index, the FPI sequentially isolates different wavelengths (though multiple wavelengths may pass at a given mirror spacing), making it well-suited for HSI. The captured FPI-tuned spectral images need to be reconstructed to obtain spectral values for each wavelength. The target consists of small amounts of erbium oxide (Er_2_O_3_), referred to as the EO target in this paper. It exhibits sharp absorption spikes at specific wavelengths, notably at 490 nm, 522 nm, 654 nm, and 800 nm, which correspond to the energy gaps between the ground state and various excited states of Er^3+^ [19,20].

Two spectra were recorded under illumination from a xenon (Xe) light source (Dyonics 300XL Xenon Light, Smith & Nephew, Inc., London, UK) and a tungsten halogen (TH) light source (SLS201, Thorlabs, Inc., Newton, NJ, USA). For our HSI system, data were collected at 2 nm intervals based on camera specifications. To ensure a valid comparison, the spectral resolution of the reference data was also downsampled to 2 nm. National Institute of Standards and Technology (NIST) traceable reference spectra for the targets provided by the manufacturer were used for result comparison.

Reflectance was calculated from the following equation [4,21]:

(1) $$R(x,y,\lambda )=\frac{I(x,y,\lambda )-I_{dark}(x,y,\lambda )}{I_{w}(x,y,\lambda )-I_{dark}(x,y,\lambda )}$$

where R(x,y,λ) represents the reflectance at a specific spatial position (x,y) and wavelength (λ). I, $I_{w}$, and $I_{dark}$ denote the intensities of the spectral signals measured from the target, the white reference signals measured using a white reflectance target, and the dark signals recorded without illumination (e.g., with the camera cap in place).

Since $I_{w}$ depends on the experimental condition, and $I_{dark}$ can be influenced by the camera temperature and exposure time [4], they were measured immediately before capturing I with the same settings. A NIST-traceable Spectralon reflectance target (SRT-99-100, Labsphere, Inc., North Sutton, NH, USA) with a nominal reflectance of 99% across a wide spectral range was used to measure $I_{w}$. $I_{dark}$ was measured with the camera cap in place. Spectral reflectance values R were calculated based on the average signal from a 200 × 200-pixel region at the image center to minimize noise variation; thus, the variables *x* and *y* in Equation (1) can be ignored. Furthermore, data were acquired three times for each measurement, all processing was performed on the averaged data from three measurements, and no smoothing or denoising algorithms were applied to the reflectance spectra. The camera and light sources were turned on one hour before measurements to minimize the effect of warm-up time, and all measurements were conducted in a dark room. Camera exposure time was adjusted to achieve a high intensity while avoiding saturation. The reflectance spectra, together with the reference spectrum provided by the target manufacturer, were normalized using different methods. The capability of these methods to ensure robust, standardized assessment of HSI camera performance was evaluated by comparing the measured spectra with the reference spectrum.

### 2.2. Normalization Methods

Most of these normalization methods are based on a linear transformation (scaling and/or shifting), while a few are based on nonlinear transformations. This study focuses on the following methods grouped by their underlying mathematical framework, with Section 2.2.1, Section 2.2.2 and Section 2.2.3 consisting of linear transformations and Section 2.2.4 employing nonlinear transformations.

#### 2.2.1. Maximum and Minimum Reflectance Methods

The most common normalization method is to scale an entire spectrum by dividing by the maximum reflectance (max(*R*)), ensuring that the normalized spectrum has a maximum value of one [12,16,22]. This is referred to as the Max method in this paper. If the reflectance spectrum without normalization is defined as R and the normalized spectrum as $R^{\prime }$, the Max method equation is expressed as:

(2) $$R^{\prime }=\frac{R}{max(R)}$$

To further confine the normalized spectrum within the range of [0, 1], both the minimum and maximum *R* values (min(R) and max(*R*)) can be used for shifting and scaling [15,16]. This approach is referred to as the MinMax method in this paper, and the equation is as follows:

(3) $$R^{\prime }=\frac{R-min(R)}{\max\left(R\right)-\min\left(R\right)}$$

#### 2.2.2. Full-Spectrum Reflectance Methods

Instead of a linear transformation based on one or two specific *R* values, the spectrum can be scaled based on a composite parameter derived from the *R* values at all wavelengths. Four of the evaluated methods fall into this category: area under the curve (AUC), sum of the signal (Sum), average of the signal (Ave), and L2 norm (L2) [12,16,23,24].

The equation describing the AUC method can be written as:

(4) $$R^{\prime }=\frac{R}{Area\left(R\right)}$$

where Area(R) is the area under the *R* spectrum, often calculated using the trapezoidal rule (i.e., dividing the whole area into many trapezoids and summing the areas of all the trapezoids).

The Sum method is given by the following equation:

(5) $$R^{\prime }=\frac{R}{\sum_{i=1}^{N}R_{i}}$$

where i is the index number for *R* values (value at each small band or available wavelength) and *N* is the total number of data points in the spectral arrays of *R* and *R*′.

The Ave method is expressed as:

(6) $$R^{\prime }=\frac{R}{\frac{\sum_{i=1}^{N}R_{i}}{N}}$$

For the L2 method, the spectrum is divided by the Euclidian distance of the spectrum [25]:

(7) $$R^{\prime }=\frac{R}{\sqrt{\sum_{i=1}^{N}R_{i}^{2}}}$$

#### 2.2.3. Zero-Mean Full-Spectrum Reflectance Methods

This group of methods is a subset of those described in Section 2.2.2, all of which are based on the *R* values across the entire spectrum. The standard normal variate method (SNV), also known as the z-score or standard score method, shifts the *R* spectrum to have a zero mean by subtracting the mean *R* (i.e., μ(R)) and then scales it by dividing the standard deviations (σ(R)). It measures how many σ(R) the shifted *R* values are from the zero mean. This method is typically used to reduce the impact of scattering, which can cause baseline shifts and intensity scaling that distort spectral data. The SNV normalized *R* has a zero μ(R) and a unit σ(R) [14,15]. The method standardizes the data, allowing for comparison across different datasets, even if they have varying μ(R) and σ(R). The formula for the SNV method is defined as [8]:

(8) $$R^{\prime }=\frac{R-\mu (R)}{\sigma (R)}$$

A variant of the SNV method is known as Pareto normalization, where the zero-mean reflectance is divided by $\sqrt{\sigma (R)}$ instead of σ(R), as in the SNV method. This method reduces the influence of high-variance signals in the normalized outcome [17,26,27]. The Pareto method can be described as:

(9) $$R^{\prime }=\frac{R-\mu (R)}{\sqrt{\sigma (R)}}$$

#### 2.2.4. Non-Linear Transformation Methods

Several non-linear transformation approaches exist, including log and power transformation [28]. Both reduce large values in the dataset more significantly than small values, thereby decreasing the differences between them. In this paper, we evaluate only the centering power (CP) normalization method, which applies a power transformation to the *R* spectrum followed by shifting the spectrum to have a mean of zero. This method is used to reduce variance in the spectral data [14] and reduce the impact of high-magnitude noise present in the signal. It is described by the following formula:

(10) $$R^{\prime }=\sqrt{R}-\mu (\sqrt{R})$$

### 2.3. Uniform Scaling Approach

Direct comparison of different normalization methods is challenging due to their varying scales. To address this issue, we propose a uniform scaling approach, similar to the MinMax method for a single spectrum, to further process each set of R or $R^{\prime }$ spectra. Unlike the MinMax method applied to an individual spectrum, the uniform scaling approach identifies the minimum and maximum values across the entire set of spectra to scale each spectrum using the same minimum and maximum values, ensuring that the relative positions of these spectra with respect to each other are preserved.

### 2.4. Performance Evaluation Metrics

To evaluate the performance of the normalization methods, both the measured R and $R^{\prime }$ spectra were compared against the NIST traceable R and $R^{\prime }$ reference spectra ($R_{NIST}$ for non-normalized data provided by the manufacturer and $R_{NIST}^{\prime }$ for normalized $R_{NIST}$). The performance of these methods was assessed using several metrics: bias (*Bias*), bias standard deviation ($\sigma_{Bias}$), root mean square error (*RMSE*), and Pearson correlation coefficient (r). The equations for evaluating the $R^{\prime }$ spectra using these metrics are provided below. For the *R* spectra, the equations are similar, except that R′ and $R_{NIST}^{\prime }$ are replaced with R and $R_{NIST}$, respectively.

(11) $$Bias=\frac{\sum_{i=1}^{N}\left(R_{i}^{\prime }-R_{NIST,i}^{\prime }\right)}{N}$$

(12) $$\sigma_{Bias}=\sqrt{\frac{\sum_{i=1}^{N}{\left[\left(R_{i}^{\prime }-R_{NIST,i}^{\prime }\;\right)-Bias\right]}^{2}}{N-1}}$$

(13) $$RMSE=\sqrt{\frac{\sum_{i=1}^{N}{\left(R_{i}^{\prime }-R_{NIST,i}^{\prime }\right)}^{2}}{N}}$$

(14) $$r=\frac{\sum_{i=1}^{N}\left(R_{i}^{\prime }-\mu (R^{\prime }))(\;R_{NIST,i}^{\prime }-\mu (R_{NIST}^{\prime })\right)}{\sqrt{\sum_{i=1}^{N}{\left(R_{i}^{\prime }-\mu (R^{\prime })\right)}^{2}}\sqrt{\sum_{i=1}^{N}{\left(R_{NIST,i}^{\prime }-\mu (R_{NIST}^{\prime })\right)}^{2}}}$$

where i is the index number for $R^{\prime }$ values and N is the total number of $R^{\prime }$ values.

## 3. Results

We collected the *R* spectra of the EO target in the 450–830 nm range under illumination from the Xe and TH light sources. The metrics of *Bias*, $\sigma_{Bias}$, *RMSE*, and r were used to quantitatively assess and compare the reference spectra with the collected spectra across different normalization methods.

### 3.1. Comparison of Normalization Methods

**Figure 1:**
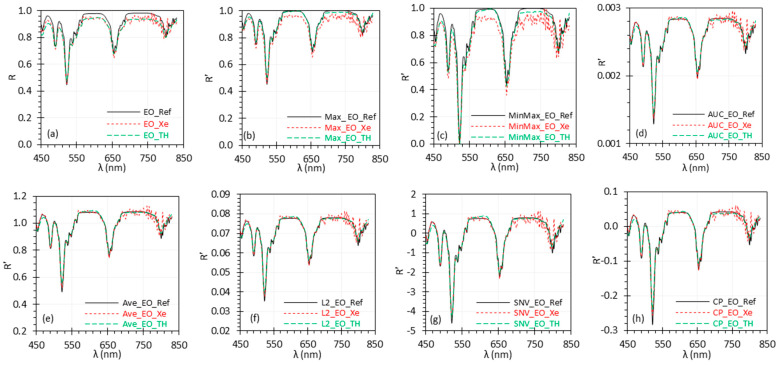
*R* (**a**) and *R*′ (**b**–**h**) spectra of the EO target. Each *R*′ legend label (e.g., “Ave_EO_Ref”) contains three parts, separated by an underscore mark. The first part indicates the normalization method (e.g., Ave, SNV). The middle part specifies the target (i.e., EO). The last part indicates whether the spectrum is the reference (Ref) or measured under illumination by the Xe or TH source. The *R* legend labels only have the last two parts of the *R*′ legend labels.

Figure 1a shows the *R* spectra from the EO target, highlighting the deviation of the measured *R* spectra from the reference *R* spectrum. Figure 1b–h presents the $R^{\prime }$ spectra using seven of the nine normalization methods—Max, MinMax, AUC, Ave, L2, SNV, and CP—as described by Equations (2), (3), (4), (6), (7), (8), and (10), respectively. Due to space constrains, the results for the Sum and Pareto methods are not shown, as they closely resemble the AUC and SNV methods, exhibiting nearly identical relative spectral shapes. Readers can refer to the Data Availability Statement for additional results.

The figure indicates that different normalization methods yield spectra on varying scales, making cross-comparison challenging. Additionally, the SNV, Pareto, and CP normalization methods produce zero-centered spectra, complicating result interpretation. Overall, the figure shows that, except for Max and MinMax normalization with the Xe light data, all other methods reduce the deviation between the measured spectra and the reference spectrum.

Table 1 quantifies the differences and relationships between the measured spectra and the reference spectrum (most of which are shown in Figure 1) using various metrics. The r values remained unaffected by the linear normalization methods but changed for the nonlinear CP normalization method. However, due to the differing scales of the *R*′ spectra, it is challenging to quantitatively compare these data in the table to determine the most suitable normalization method for this dataset.

For example, the Max and MinMax methods yield measured *R*′ spectra that deviate considerably from the reference *R*′ spectrum (Figure 1b,c), while the deviations are notably smaller with the SNV method (Figure 1g). Yet, the *RMSE* values for the Max and MinMax methods are significantly smaller than the value for the SNV method. Additionally, the *R*′ values derived from the AUC and Sum methods are very small, often rounding to zero at three decimal places, as these methods scale the *R*′ spectra down significantly. In summary, quantitative error measurement and comparison can be misleading if the two sets of spectra are on significantly different scales. This highlights the importance of applying a second-stage normalization (i.e., uniform scaling, as discussed in Section 2.3 and Section 3.2) to ensure fair comparisons between different normalization methods.

### 3.2. Uniform Scaling for Each Set of R or R′ Spectra

**Figure 2:**
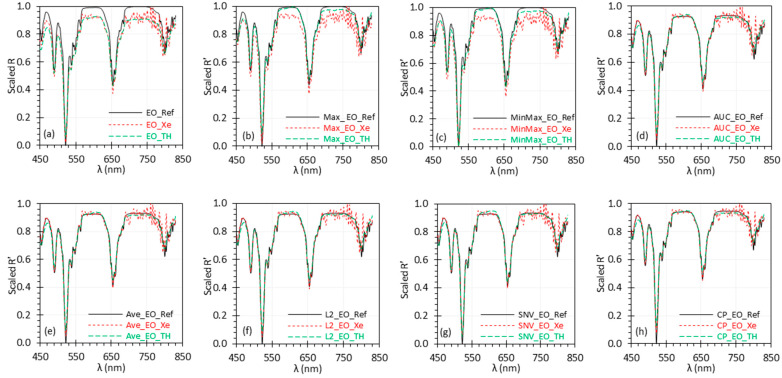
Uniformly scaled *R* (**a**) and *R*′ (**b**–**h**) spectra of the EO target in Figure 1.

To properly compare different normalization methods, we applied the uniform scaling approach (Section 2.3) to each set of R or R′ spectra discussed in Section 3.1, ensuring that all sets were adjusted to the same range of [0, 1]. The uniformly scaled results are shown in Figure 2 and Table 2. The trends in Figure 2 are consistent with those observed in Figure 1. Like Table 1, Table 2 quantifies the differences and relationships between the measured spectra and the reference spectrum (most of which are shown in Figure 2) after uniform scaling, using various metrics.

Similar to Figure 1, Figure 2 clearly shows that the measured scaled *R* spectra deviate from the reference scaled *R* spectrum. Upon applying normalization, the measured scaled *R*′ spectrum under the TH light exhibited a significant reduction in deviation from the reference scaled *R*′ spectrum across all normalization methods, as indicated by the reduced *Bias* and *RMSE* values in Table 2. However, while most normalization methods reduced the deviation between the measured scaled *R*′ and the reference scaled *R*′ under the Xe light, the Max and MinMax methods still showed higher deviation due to noise in the spectra beyond 650 nm, with *Bias* and *RMSE* values comparable to the scaled *R* data. The r remained unaffected by linear normalization methods or uniform scaling approach across light sources, except for the nonlinear CP normalization. The SNV method was slightly better in terms of *RMSE* and $\sigma_{Bias}$ than the other methods. Overall, all normalization methods demonstrated good performance, except for the Max and MinMax methods, which were less effective in the presence of outliers or high noise in the spectra. After uniform scaling, consistent conclusions can be drawn from the reflectance spectra and the quantitative metrics regarding the performance of the normalization methods studied.

**Figure 3:**
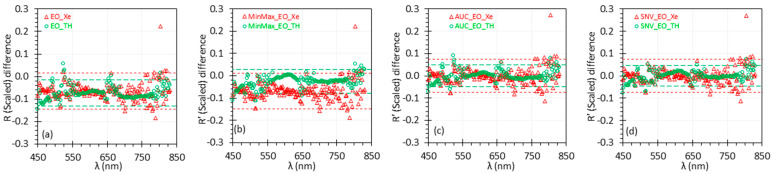
Difference plots of uniformly scaled reflectance spectra. The red and green dashed lines represent the *LoA* for Xe and TH, respectively. (**a**) Plot for scaled *R*; (**b**–**d**) plot for scaled *R*′ using the MinMax, AUC, and SNV methods.

Figure 3 shows difference plots for the scaled *R* and *R*′ spectra normalized using three representative methods followed by a second-step uniform scaling with wavelength on the x-axis. The reflectance difference can also be plotted against the mean reflectance value following a similar approach to traditional Bland–Altman plots. For additional plots, including those with the reflectance value on the x-axis, please refer to the Data Availability Statement.

The upper and lower limits of agreement (*LoA* = ${Bias\pm 1.96\;\sigma }_{Bias}$) for the 95% confidence interval are indicated by dashed lines for each dataset. These results align with the analyses of Figure 2 and Table 2. The AUC and SNV normalization methods performed well, showing a *Bias* near zero and narrower *LoA* compared to the non-normalized data. While the MinMax method slightly improved the TH data, it did not improve the Xe data. Overall, the Xe results displayed a wider range of *LoA* than the TH results across all graphs, primarily due to the higher noise level in the Xe spectrum, particularly at wavelengths beyond 650 nm.

## 4. Discussion

In this study, we applied various normalization methods to reflectance data measured using two different light sources or from the target manufacturer. These methods primarily involved linear transformations, except for the CP normalization, which used a non-linear transformation. Additionally, we developed a uniform scaling approach to ensure that all spectral sets could be compared on the same scale. The measured reflectance spectra were compared to the reference spectrum to assess how effectively the methods improved spectral accuracy by reducing experimental variability and providing more robust reflectance spectra across different measurement conditions, such as varying illuminations.

### 4.1. Effect of Normalization Methods on Different Calibration Targets

**Figure 4:**
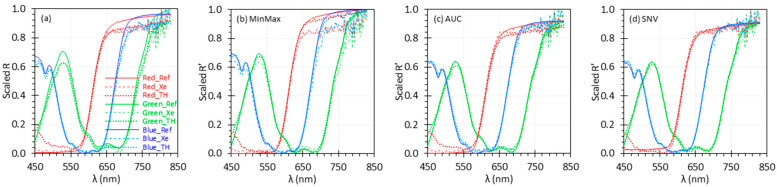
Uniformly scaled *R* (**a**) and *R*′ (**b**–**d**) spectra of three targets. *R*′ spectra were normalized using the MinMax (**b**), AUC (**c**), and SNV (**d**) methods.

Biological tissues typically contain various peaks and can have varying baselines, which makes overall spectra complex in nature [29]. To replicate some of these complexities, we utilized the EO calibration target in this study. To cover a broad range of target/tissue types, we also analyzed red, green, and blue diffuse reflectance calibration targets (SCS-RD-010, SCS-GN-010, and SCS-BL-010, Labsphere, Inc., North Sutton, NH, USA). Figure 4 shows uniformly scaled *R* and *R*′ spectra of the three targets, similar to the approach shown in Figure 2. The complete dataset, which includes all the normalization methods described in this study and the uniform scaling results, is provided in the supplementary Excel file (see Supplementary Materials). In data measured using the TH light source for the red color calibration target, we observed some errors at shorter wavelengths, specifically below 550 nm, due to the low intensity of the TH source and the red target’s low reflectance in this range. Across all targets, most normalization methods consistently reduced deviations from the reference spectrum. Again, the MinMax method displayed increased deviation between the Xe and reference spectra because of high noise. The SNV method slightly outperformed the AUC method in minimizing the difference between the measured and reference spectra.

### 4.2. Potential Challenges with Normalization

Normalization can remove the effects from external factors in the measured reflectance data. However, a concern arises that normalization might also eliminate a target’s or tissue’s inherent features. Biological tissues often exhibit peaks or features that vary across the entire spectral range or at specific wavelengths based on tissue properties [30,31,32,33,34]. To classify tissues based on their optical characteristics, it is important to preserve these inherent variations. While normalization can be an important step for reducing the influence of external factors, it is equally important to assess whether it also diminishes the inherent contributions of tissue constituents.

**Figure 5:**
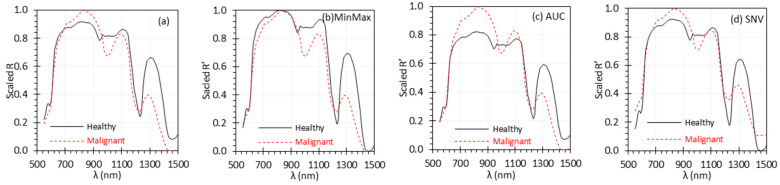
Uniformly scaled *R* (**a**) and *R*′ (**b**–**d**) spectra of ex vivo breast tissues. The *R* data were extracted from [30] by converting graphical spectra into digital data. *R*′ spectra were normalized using the MinMax (**b**), AUC (**c**), and SNV (**d**) methods.

To address this potential concern, we applied normalization to spectra obtained from three previously published articles [30,31,35], where reflectance was measured using a diffuse reflectance spectroscopy system with the probe in contact with the tissue. Since these results are based on contact measurements, they are expected to be less susceptible to variations in measurement conditions, such as inconsistent light illumination, which could otherwise affect the measurement accuracy. Figure 5a–d (based on [30]) shows that even after normalization, it is possible to differentiate between tissue types, indicating that normalization does not diminish the inherent differences in these tissues. In another study [31], although the spectral shapes were similar, the normalized reflectance still showed significant distinctions between tissue types (not shown in this paper; see Data Availability Statement), reiterating the idea that normalization does not eliminate tissue features.

**Figure 6:**
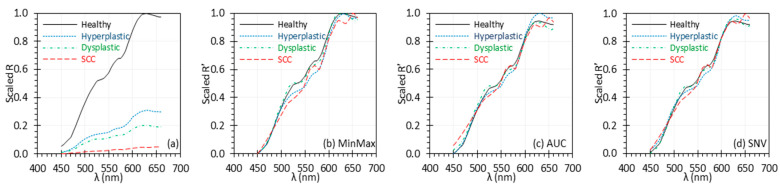
Uniformly scaled *R* (**a**) and *R*′ (**b**–**d**) spectra of oral tissues. The *R* data were extracted from [35] by converting graphical spectra into digital data. *R*′ spectra were normalized using the MinMax (**b**), AUC (**c**), and SNV (**d**) methods. (SCC: squamous cell carcinoma).

There are two special scenarios where normalization might not be recommended. The first occurs when a set of spectra have similar shapes and slopes across the entire spectral range, with nearly consistent differences in their absolute values throughout (i.e., the spectra will overlap with a shift). The second is when spectra have similar shapes but different slopes across the spectral range. The first scenario does not exist in tissues, so we focused on assessing the second scenario. Figure 6a shows a set of uniformly scaled *R* spectra for tissues at different disease stages. All spectra began with similar low *R* values that gradually increased with wavelength, with varying slopes [35]. Applying normalization in this scenario significantly diminished the inherent differences between spectra at different disease stages (Figure 6b–d), making it difficult to distinguish between them. This scenario may not be suitable for normalization, as it risks removing key contributions from the target itself, rather than external factors. However, such scenarios are rare in benchtop setups and expected to be infrequent in clinical applications, making it less likely that normalization will obscure inherent spectral variations. Nevertheless, the need for normalization and the choice of method should be determined based on the actual spectra.

### 4.3. Optimal Normalization Methods

Our study demonstrated that normalization could improve signal quality by removing the influence of external factors in most cases. Based on the performance metrics mentioned in this study, normalization reduces the error/deviation with respect to the reference spectrum; however, methods such as Max and MinMax can be performed poorly with spectra exhibiting high noise, especially at extreme reflectance values (e.g., the Xe spectra in Figure 2b,c). This is because these methods rely on a single maximum (and minimum) value to calculate the normalization factors of max(*R*) and min(*R*) in Equations (2) and (3), making them more susceptible to noise.

That said, the Max and MinMax methods can work as well as other methods with certain modifications to address noise or outliers. We refer to these modified approaches as the Mod_Max and Mod_MinMax methods. One modification involves using the average of a certain percentage (e.g., 5–10%) of the highest and lowest reflectance values as max(*R*) and min(*R*) in Equations (2) and (3). Another approach is to use the reference values at certain percentiles as max(*R*) and min(*R*). Our further investigation shows that both approaches can significantly improve the performance of these methods. The percentage or percentile values can be flexible, and a wide range of values can work effectively. There are still several other modifications that could be considered based on the spectral information. For instance, biological tissues typically exhibit several distinct absorption peaks [29]. Normalization methods based on the intensity at a defined peak [12] could be used as max(*R*) based on the spectra under contention, particularly for fluorescence spectra, where the expected peak wavelength is known.

**Figure 7:**
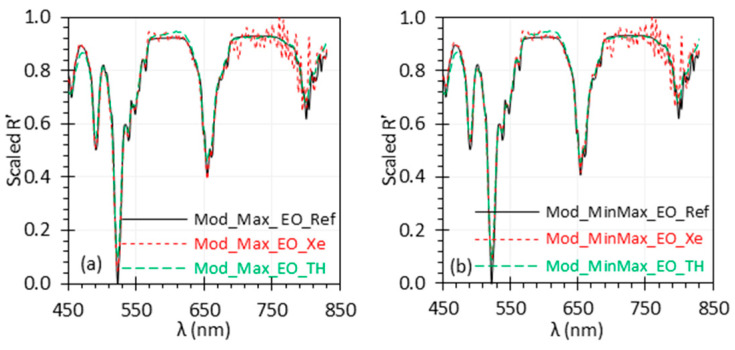
Uniformly scaled *R*′ spectra of the EO target based on the Mod_Max (**a**) and Mod_MinMax (**b**) methods.

Figure 7 presents the results using the Mod_Max and Mod_MinMax method with 20th and 80th percentile reflectance values as max(*R*) and min(*R*) for normalization. The results are comparable to those obtained using other methods (Figure 2d–h). Both the Mod_Max and Mod_MinMax methods reduced the deviation from the reference spectrum more significantly than the Max and MinMax methods (Figure 2b,c).

### 4.4. Study Limitations and Future Works

This investigation focused on spectral data measured by an HSI camera under two different light sources. When capturing target reflectance spectra, we tried to maintain consistent measurement conditions, such as working distance, illumination angle, and exposure time, but we did not study the effectiveness of normalization in mitigating these factors. Several characteristics of HSI cameras, such as spectral range, spectral response function (SRF), and the number of spectral bands/channels, can influence performance [36]. However, this study did not examine these factors or their impact on normalization and camera performance.

While we introduced modified versions of commonly used normalization methods to handle outliers or noise in Section 4.3, we did not quantify noise levels or comprehensively explore outlier or noise removal techniques, which are often applied as preprocessing steps in spectral data analysis. For example, methods that remove data whose distance from the mean exceeds three standard deviations are commonly used to improve data reliability before normalization [37]. Applying such outlier removal techniques could potentially enhance the performance of normalization methods but also risks removing useful spectral features.

In future studies, we will examine various operational and external factors—such as exposure time, warm-up time, camera focus, working distance, and illumination/target angles—that may influence spectral accuracy. Additionally, we aim to explore how normalization methods can enhance outcomes, mitigate the effects of these operational and external factors, and propose best practices for achieving robust and reliable results. Additionally, we will integrate the HSI camera into an endoscopy system to examine how external factors impact hyperspectral endoscopy and how normalization methods can help reduce the effects of these factors.

### 4.5. Summary

In this study, we evaluated the performance of various normalization methods in mitigating the impact of external factors on reflectance spectra, highlighting normalization as a crucial preprocessing step for HSI camera evaluation. Previous studies have directly compared normalization methods, which can be misleading due to the differing scales of normalized spectra. We proposed a second-step uniform scaling approach to address this issue by standardizing the range of all normalized spectra, enabling direct comparison without the confounding effects of scale differences. This approach preserves the relative positions and relationships among normalized spectra, enhancing the robustness and interpretability of spectral data. It also avoids the negative values generated by some normalization methods (e.g., SNV), allowing for more flexible image formats when saving the normalized hypercubes, if needed.

For HSI camera evaluations, reflectance spectra are influenced by factors such as light source age, working distance, incident angle, and warm-up time. Even under controlled conditions, spectra from the same target using the same camera may deviate from the reference over time due to these experimental factors, rather than inherent target properties. Therefore, normalization is essential to accurately assess spectral performance by minimizing these deviations, ensuring that comparisons focus on target characteristics rather than external influences.

For the reflectance spectra of the EO target, a variety of normalization methods performed equally well, a conclusion differing from previous studies, largely because we applied the second-step uniform scaling approach. However, for noisy spectra, the Max and MinMax methods, which rely on one or two specific reflectance values for normalization, can produce erroneous results, highlighting the need for careful selection of normalization methods. The Max and MinMax methods can, however, be modified to mitigate noise and achieve performance comparable to other methods. Similarly, other normalization methods can be modified as well. For example, to mitigate the impact of noise or outliers, median values could be used in methods like SNV or CP normalization instead of mean values.

Overall, the performance differences between most normalization methods were minimal for the spectra we measured except for the Max and MinMax methods. Methods based on linear transformations of *R* values across the entire spectrum (such as AUC, Sum, Ave, L2, SNV, and Pareto) were robust against outliers and noise since they account for *R* values at all the wavelengths to calculate a composite outcome. Among these, the SNV method showed slightly better performance than the others for the spectra we evaluated (see Data Availability Statement).

Finally, the decision to normalize a set of spectra and the choice of normalization method will vary depending on the spectral quality and content as well as the algorithm used to further analyze the normalized spectra. In addition to spectral and performance evaluation metrics, other parameters, such as classification accuracy, can also be used to assess the impact of normalization [13,17,37]. However, the results may vary depending on the algorithms applied. Consequently, the performance evaluation metrics for comparing these methods should be selected based on empirical analyses, highlighting its importance in guiding the selection process. As mentioned in Section 4.2, in rare cases such as where spectra have similar shapes but different slopes across the spectral range (Figure 6), normalization can remove features inherent to the target. In such instances, normalization may not be appropriate, even if the signal is influenced by external factors. However, in general, normalization preserves inherent spectral features while mitigating the effects of external factors (Figure 5), and thus it remains important for robust laboratory evaluation of HSI cameras.

## 5. Conclusions

In conclusion, this study underscores the importance of normalization as a critical preprocessing step for mitigating the effects of external factors on reflectance spectra measured using HSI cameras. The second-step uniform scaling approach we proposed enables direct comparisons of normalization methods by standardizing the range of normalized spectra, preserving their relative relationships, and improving the robustness of spectral analysis.

While normalization generally preserves essential spectral features, it should be applied selectively based on the specific application. For laboratory evaluation of HSI cameras, normalization helps reduce errors introduced by experimental factors, ensuring more accurate assessments of spectral performance. Although most normalization methods performed similarly, noisy spectra presented challenges for the Max and MinMax methods, highlighting the need for careful method selection and potential modification to address noise. Overall, normalization methods based on linear transformations across the entire spectrum are robust across different spectral types.

## Figures and Tables

**Table 1:** Quantitative evaluation of normalization methods (metrics for *R* or *R′*).

Metrics	*R*		*R*′, Max		*R*′, MinMax		*R*′, AUC		*R*′, Sum		*R*′, Ave		*R*′, L2		*R*′, SNV		*R*′, Pareto		*R*′, CP	
	Xe	TH	Xe	TH	Xe	TH	Xe	TH	Xe	TH	Xe	TH	Xe	TH	Xe	TH	Xe	TH	Xe	TH
*Bias*	−0.035	−0.039	−0.035	−0.005	−0.069	−0.025	0.000	0.000	0.000	0.000	0.000	0.000	0.000	0.000	0.000	0.000	0.000	0.000	0.000	0.000
$\sigma_{Bias}$	0.022	0.016	0.022	0.015	0.041	0.027	0.000	0.000	0.000	0.000	0.024	0.016	0.002	0.001	0.220	0.137	0.069	0.045	0.012	0.009
*RMSE*	0.041	0.042	0.042	0.016	0.080	0.037	0.000	0.000	0.000	0.000	0.024	0.016	0.002	0.001	0.220	0.137	0.069	0.045	0.012	0.009
*r*	0.976	0.991	0.976	0.991	0.976	0.991	0.976	0.991	0.976	0.991	0.976	0.991	0.976	0.991	0.976	0.991	0.976	0.991	0.978	0.991

Note: Red numbers indicate the worst results in this table.

**Table 2:** Quantitative evaluation of normalization methods after uniform scaling (metrics for uniformly scaled *R* or *R*′).

Metrics	Scaled *R*		Scaled *R*′, Max		Scaled *R*′, MinMax		Scaled *R*′, AUC		Scaled *R*′, Sum		Scaled *R*′, Ave		Scaled *R*′, L2		Scaled *R*′, SNV		Scaled *R*′, Pareto		Scaled *R*′, CP	
	Xe	TH	Xe	TH	Xe	TH	Xe	TH	Xe	TH	Xe	TH	Xe	TH	Xe	TH	Xe	TH	Xe	TH
*Bias*	−0.065	−0.073	−0.064	−0.010	−0.069	−0.025	0.000	0.000	0.000	0.000	0.000	0.000	0.000	0.001	0.000	0.000	0.000	0.000	0.000	0.000
$\sigma_{Bias}$	0.041	0.030	0.041	0.027	0.041	0.027	0.038	0.025	0.038	0.025	0.038	0.025	0.038	0.025	0.038	0.024	0.038	0.025	0.034	0.025
*RMSE*	0.076	0.079	0.076	0.028	0.080	0.037	0.038	0.025	0.038	0.025	0.038	0.025	0.038	0.025	0.038	0.024	0.038	0.025	0.034	0.025
*r*	0.976	0.991	0.976	0.991	0.976	0.991	0.976	0.991	0.976	0.991	0.976	0.991	0.976	0.991	0.976	0.991	0.976	0.991	0.978	0.991

Note: Red numbers indicate the worst results in this table.

## Data Availability

The complete datasets, including all the relevant figures and tables in this paper, as well as additional data omitted due to space limitations, are provided as Supplementary Material in an Excel file.

## Supplementary Materials

The following supporting information can be downloaded at: https://www.mdpi.com/article/10.3390/bios15010020/s1, accessed on GitHub at https://github.com/qz-w/Normalization-of-HSI-spectra.git (Accessed on 25 December 2024).
